# A Lightweight Cross-Gated Dual-Branch Attention Network for Colon and Lung Cancer Diagnosis from Histopathological Images

**DOI:** 10.3390/medsci13040286

**Published:** 2025-11-26

**Authors:** Raquel Ochoa-Ornelas, Alberto Gudiño-Ochoa, Sergio Octavio Rosales-Aguayo, Jesús Ezequiel Molinar-Solís, Sonia Espinoza-Morales, René Gudiño-Venegas

**Affiliations:** 1 Systems and Computation Department, Instituto Tecnológico de Ciudad Guzmán, Tecnológico Nacional de México, Ciudad Guzmán 49100, Jalisco, Mexico; sergio.ra@cdguzman.tecnm.mx; 2 Centro Universitario de Ciencias Exactas e Ingenierías (CUCEI), Electronics and Computing Division, Universidad de Guadalajara, Guadalajara 44430, Jalisco, Mexico; gudino.ochoa.alberto@gmail.com; 3 Electronics Department, Instituto Tecnológico de Ciudad Guzmán, Tecnológico Nacional de México, Ciudad Guzmán 49100, Jalisco, Mexico; jesus.ms@cdguzman.tecnm.mx; 4 Instituto Tecnológico de Tepic, Tecnológico Nacional de México, Tepic 63175, Nayarit, Mexico; sespinoza@ittepic.edu.mx; 5 Instituto Tecnológico de Ciudad Guzmán, Tecnológico Nacional de México, Ciudad Guzmán 49100, Jalisco, Mexico; rene.gv@cdguzman.tecnm.mx

**Keywords:** lung cancer, colon cancer, artificial intelligence, histopathological images

## Abstract

Background/Objectives: Accurate histopathological classification of lung and colon tissues remains difficult due to subtle morphological overlap between benign and malignant regions. Deep learning approaches have advanced diagnostic precision, yet models often lack interpretability or require complex multi-stage pipelines. This study aimed to develop an end-to-end dual-branch attention network capable of achieving high accuracy while preserving computational efficiency and transparency. Methods: The architecture integrates EfficientNetV2-B0 and MobileNetV3-Small backbones through a cross-gated fusion mechanism that adaptively balances global context and fine structural details. Efficient channel attention and generalized mean pooling enhance discriminative learning without external feature extraction or optimization stages. Results: The network achieved 99.84% accuracy, precision, recall, and F1-score, with an MCC of 0.998. Grad-CAM maps showed strong spatial correspondence with diagnostically relevant histological structures. Conclusions: The end-to-end framework enables the reliable, interpretable, and computationally efficient classification of lung and colon histopathology and has potential applicability to computer-assisted diagnostic workflows.

## 1. Introduction

Cancer is a group of diseases characterized by uncontrolled cell proliferation, capable of invading surrounding tissues and spreading to distant organs through the blood and lymphatic systems [1,2]. Within this spectrum, lung and colorectal cancers are among the most prevalent and lethal worldwide. In 2022, they accounted for approximately 4.27 million new cases and 2.74 million deaths, representing one of the greatest global burdens in oncology [3,4]. Lung cancer alone caused nearly 1.8 million deaths, while colorectal cancer contributed close to one million [5,6,7]. Early and accurate detection markedly improves survival—when identified at localized stages, five-year survival can exceed 90%—yet many cases are still diagnosed only after progression [8,9].

From a histopathological standpoint, adenocarcinoma arises from glandular epithelial tissue and predominates in both organs, whereas squamous cell carcinoma originates from the squamous epithelium and is more common in the respiratory tract [10,11,12,13,14]. Less frequent variants, such as large-cell carcinoma, add diagnostic complexity [15,16]. A practical example is the difficulty of distinguishing benign colonic glands from early adenocarcinoma when glandular crowding and nuclear stratification are minimal [17].

Histopathological evaluation of hematoxylin and eosin (H&E)-stained slides remains the reference standard for determining tumor type and malignancy grade [18,19,20]. However, manual diagnosis is time-consuming and inherently subjective, particularly when pathologists must identify fine morphological cues—such as glandular distortion in colon tissue or keratinization patterns in lung carcinoma [21,22]. These limitations have motivated the adoption of artificial intelligence (AI) in computational pathology. Within this field, deep learning (DL)—especially convolutional neural networks (CNNs)—has emerged as one of the most effective paradigms for analyzing tissue architecture and cellular organization [23,24,25,26,27]. By learning discriminative texture and color representations directly from image data, CNNs have achieved performance comparable to that of experienced pathologists in specific diagnostic tasks [28,29,30,31].

Despite this progress, two major limitations persist in CNN-based histopathological analysis. First is the issue of model complexity: high-performing networks such as DenseNet, EfficientNet-V2L, and Vision Transformers contain tens of millions of parameters and require extensive computational resources, which restricts reproducibility and adoption in typical computer-aided diagnostic (CAD) frameworks [32,33,34].

Second is the issue of fragmented pipelines: many approaches separate preprocessing, feature extraction, and classification into distinct modules. This multi-stage design interrupts gradient flow and prevents joint optimization, leading to redundant computation and potential information loss between stages [35,36,37,38].

Motivated by these limitations, which represent clear gaps in current AI/DL approaches for histopathological cancer diagnosis, this study introduces a compact end-to-end dual-branch attention network for classifying colon and lung cancer images using the LC25000 dataset [39]. The model maps raw images directly to class predictions without external feature engineering or auxiliary classifiers. All layers—from early convolutional filters to the final classification head—are optimized jointly toward the same objective, improving convergence stability and overall consistency.

The proposed model integrates two complementary lightweight backbones: EfficientNetV2-B0, which captures high-level semantic information, and MobileNetV3-Small, which specializes in fine-grained local features [40,41]. Their outputs are fused through a cross-gated attention block that adaptively balances information flow between branches. The design further incorporates efficient channel attention and generalized mean pooling to enhance focus on discriminative regions while maintaining low computational cost. Together, these components yield strong representational capacity within compact architecture.

The study focuses on evaluating the representational performance and efficiency of this architecture under controlled experimental conditions. The goal is to demonstrate that a fully end-to-end lightweight design can achieve accuracy comparable to deeper, resource-intensive networks while maintaining reproducibility and simplicity in training. The main contributions are the following:

1. A fully end-to-end dual-branch attention network that unifies global and local representations without intermediate processing stages;
2. A cross-gated fusion mechanism that enhances feature complementarity between EfficientNetV2-B0 and MobileNetV3-Small while minimizing parameter overhead;
3. An empirical validation showing that high classification accuracy and low computational cost can coexist, evaluated on the LC25000 dataset of colon and lung histopathological images.

The structure of this paper is as follows. Section 2 discusses related work in histopathological image analysis. Section 3 details the architecture and methodological design of the proposed model. Section 4 reports the experimental results and analysis, and Section 5 summarizes the conclusions and future perspectives.

## 2. Related Works

Research on lung and colon histopathology classification has evolved along three main directions: (i) transfer learning with lightweight convolutional networks, (ii) hybrid frameworks combining deep and handcrafted features with optimization algorithms, and (iii) attention- or Transformer-based architectures. Most studies rely on the LC25000 dataset as a benchmark.

Several works adapt pretrained CNN backbones to distinguish adenocarcinoma, squamous cell carcinoma, and benign tissue. Using MobileNetV2, one study reported ≈ 97.6% accuracy after augmenting the dataset with slides from the U.S. National Cancer Institute (NCI) [42]. With EfficientNet-B3, five-class classification reached 99.4% after replacing about 1000 synthetic images with real NCI samples, while Grad-CAM visualizations confirmed that the network attended to diagnostically relevant regions [43]. Inception-ResNetV2 achieved 95.9% when combined with local binary patterns (LBP) [44]. These results demonstrate that transfer learning can be highly competitive, though single-backbone models remain vulnerable to domain shifts between staining protocols or institutions.

Another line of work extracts deep embeddings from CNNs and feeds them into traditional classifiers. Gowthamy et al. fused features from ResNet-50, InceptionV3, and DenseNet and trained a Kernel Extreme Learning Machine (KELM) optimized by a mutation-boosted Dwarf Mongoose algorithm, achieving 98.9% accuracy [45]. Bhattacharya et al. combined ResNet-18 and EfficientNet-B4 embeddings, selected informative subsets via a Whale Optimization Algorithm with adaptive β-Hill Climbing (AdBet-WOA), and used a support vector machine (SVM) classifier to reach 99.96% [46]. Roy et al. proposed a channel-attention feature extractor with an adaptive genetic selector followed by k-Nearest Neighbors, reporting 99.75% [47]. Despite their precision, these multi-stage schemes require separate training steps and hyperparameter tuning, which hinders joint optimization and reproducibility.

Some studies optimize entire pipelines with metaheuristic search or model committees. Mengash et al. combined MobileNet features with contrast-enhanced inputs and a Deep Belief Network, using a marine-predators algorithm (MPADL-LC3) to tune hyperparameters and reaching 99.27% [48]. AlGhamdi et al. proposed BERTL-HIALCCD, which integrates a modified ShuffleNet extractor, a deep recurrent classifier, and the Coati optimizer, reporting 99.22% [49]. A further ensemble coupled Wiener-filtered inputs with a channel-attention ResNet50 optimized by Tuna Swarm Optimization, merging outputs from an ELM, CNN, and LSTM to attain 99.6% [50]. Even higher-order fusions—such as MobileNetV2 + EfficientNetB3 features optimized via Grey Wolf Optimizer and evaluated with multiple learners—hovered near 95% [38]. While such techniques can add marginal accuracy gains, they greatly increase architectural and hyperparameter complexity.

To capture long-range dependencies without losing local detail, recent works incorporate attention mechanisms. LMVT merges a MobileViT backbone, multi-head self-attention, and convolutional block attention with texture cues from the Simple Gray-Level Difference Method and curriculum augmentation for minority classes, achieving 99.75% [51]. ViT-DCNN combined Vision Transformer self-attention with deformable convolutions and reached ≈94%, outperforming its CNN baselines [52]. A compound-scaled EfficientNetV2-L model attained ≈99.97% with Grad-CAM visualization [33]. A compact 1-D CNN with Squeeze-and-Excitation reported 100% accuracy using only 0.35 M parameters and 6.4 M FLOPs [53]. While attention modules improve contextual reasoning, they often raise memory requirements or depend on dataset-specific fine-tuning recipes.

Some CAD systems merge multiple CNN embeddings with handcrafted descriptors. For example, one approach integrates EfficientNetB0, MobileNet, and ResNet-18 features with gray-level co-occurrence matrices and LBP, applying non-negative matrix factorization and minimum-redundancy–maximum-relevance selection to achieve 99.7% [54]. Another merges pooling- and fully connected-layer features from several CNNs, compresses them via canonical correlation analysis, and uses ANOVA/χ^2^ feature selection before an SVM classifier, reaching 99.8% with only 50 variables [55]. Even outside the LC25000 dataset, Vision Transformer + XGBoost ensembles have improved colorectal carcinoma recognition after class re-balancing and DCGAN augmentation [56]. These hybrid systems exploit complementary information but remain multi-stage and not jointly trainable.

A few studies emphasize end-to-end efficiency and interpretability. A multi-scale CNN with ≈1.1 M parameters combined with Grad-CAM and SHAP explanations reached 99.2% [57]. Another explainable-AI study integrated LIME and SHAP to visualize both low- and high-level cues, obtaining ≈100% accuracy [58]. Such results highlight the importance of maintaining coherence under domain shifts and minimizing auxiliary steps that dilute the “end-to-end” principle.

Compared with single-backbone transfer approaches [38,42,43,44] and multi-stage feature-selection pipelines [45,46,47,48,49,50], our method preserves a single trainable stream, no handcrafted descriptors, external classifiers, or metaheuristic tuning. Relative to attention-heavy architectures [33,51,52,53], we pair two complementary lightweight backbones, EfficientNetV2-B0 for global semantics and MobileNetV3-Small for fine spatial detail, and fuse them through a cross-gated attention module. This design retains complementary cues within one optimization loop, reducing computational overhead and fragmentation while sustaining state-of-the-art accuracy on LC25000.

## 3. Proposed Model

This section presents the design and experimental framework of the proposed BiLight-Attn-LC architecture for lung and colon cancer histopathological image classification. It begins with a description of the dataset used for model training and evaluation, followed by the three main architectural components: dual-branch lightweight fusion, cross-gated feature integration, and a hybrid spatial–channel attention descriptor. The section also outlines the training configuration based on the AdamW optimizer with a Warmup–Cosine learning schedule to ensure stable convergence, and the use of Grad-CAM saliency maps to visualize the morphological regions that drive model decisions.

### 3.1. Dataset Used

The experiments were conducted on the LC25000 dataset, a publicly available benchmark containing 25,000 histopathological image tiles evenly distributed across five diagnostic classes. These images are not whole-slide images (WSI); instead, each sample is a 768 × 768 pixel cropped tile provided in JPEG format. This format is typical for LC25000-based studies and does not involve the gigabyte-scale storage or multi-resolution pyramidal structures associated with WSI.

The dataset was originally developed by Borkowski et al. [39] from HIPAA-compliant, pathologist-validated histopathological samples comprising 1250 original images: 750 from lung tissue (250 benign, 250 adenocarcinomas, and 250 squamous cell carcinomas) and 500 from colon tissue (250 benign and 250 adenocarcinomas). The dataset’s current version includes 25,000 augmented images, as released by its authors through the LC25000 repository, to ensure balanced representation and morphological diversity across classes. Each class contains 5000 images, summarized in Table 1.

**Figure 1:**
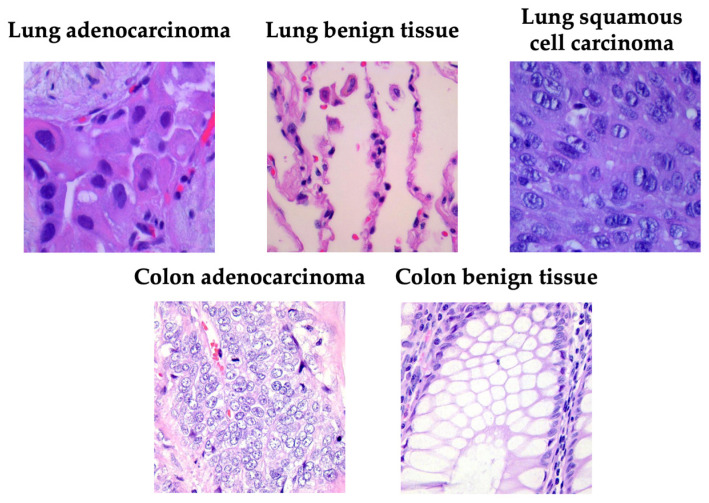
Examples of samples taken from the LC25000 dataset.

Figure 1 presents a representative image from each category, illustrating the visual heterogeneity of the LC25000 dataset in terms of color distribution, glandular architecture, and cellular density.

### 3.2. Model Architecture

**Figure 2:**
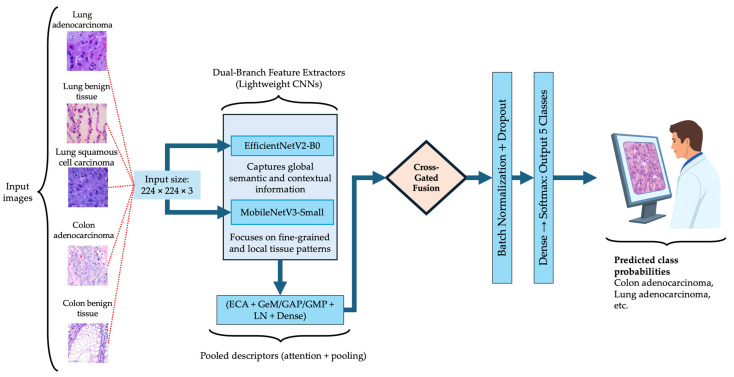
Overall architecture of the proposed BiLight-Attn network.

The proposed BiLight-Attn-LC network is an end-to-end dual-branch convolutional model designed to integrate global semantic context and local textural features from histopathological images [59]. The model takes an input image $x\in R^{H\times W\times 3}$ and outputs a class probability vector $\hat{y}\in R^{C}$:

(1) $$\hat{y}=f_{\theta }\left(x\right)=Softmax\left(W_{c}\Phi \left(x\right)+b_{c}\right)$$

where Φ(x) denotes the feature representation after dual-branch fusion, and $W_{c},b_{c}$ are the classification head parameters. The architecture comprises three sequential modules: dual-branch lightweight feature extraction, cross-gated fusion, and hybrid descriptor and classification head. Figure 2 illustrates the overall BiLight-Attn-LC architecture. All images from the LC25000 dataset were resized from their original 768 × 768 resolution to 224 × 224 to comply with the input requirements of EfficientNetV2-B0 and MobileNetV3-Small. The following subsections describe each component of the architecture in detail.

#### 3.2.1. Dual-Branch Lightweight Fusion

The model incorporates two complementary lightweight encoders: EfficientNetV2-B0 and MobileNetV3-Small. These architectures were selected for their favorable balance between representational capacity and computational efficiency, and both have demonstrated strong generalization performance in histopathological image classification tasks, where accuracy–efficiency trade-offs are critical [33,35,38]. With an input resolution of 224 × 224, EfficientNetV2-B0 has approximately 7.2 million parameters and 1457.7 million FLOPs per image, while MobileNetV3-Small contains 2.6 million parameters and 119.2 million FLOPs.

For comparison, a larger variant such as EfficientNetV2-L contains approximately 119.0 million parameters and 24,618.7 million FLOPs. This means that the chosen EfficientNetV2-B0 backbone reduces the parameter count and the computational cost by more than 90% relative to EfficientNetV2-L, while still providing strong feature extraction capability.

Let $f_{A}(x)\in R^{h_{A}\times w_{A}\times c_{A}}$ and $f_{B}(x)\in R^{h_{B}\times w_{B}\times c_{B}}$ denote the feature maps extracted by each backbone:

(2) $$f_{A}(x)=EfficientNetV2B0(x),\;\;\;f_{B}(x)=MobileNetV3Small(x)$$

where $f_{A}(x)$ captures global and contextual information, while $f_{B}(x)$ focuses on fine-grained structures such as glandular boundaries or nuclear arrangements.

Each branch output passes through a pooled descriptor that combines efficient channel attention (ECA) [60], and three complementary pooling operations generalized mean (*GeM*), average (*GAP*), and max (*GMP*), to produce a compact embedding:

(3) $$d_{i}=\;LN({Dense}_{GELU}([GeM(f_{i}),GAP(f_{i}),GMP(f_{i})])),\;\;\;i\in \left\{A,B\right\}$$

Here, *LN* denotes layer normalization, and the dense projection maps each descriptor to a shared latent space of dimension D=128.

The combination of pooling operations captures both global activation trends (via *GAP*) and localized high-intensity responses (via *GMP*), while *GeM* provides a smooth interpolation between them [61,62]. To emphasize informative channels, an ECA block applies a 1D convolution of kernel size k=5 over the globally averaged feature vector:

(4) $$w=\sigma (Conv1D_{k}(GAP(f_{i}))),\;\;\;\;\;\;\tilde{fi}=fi⊙w$$

where σ denotes the sigmoid activation and ⊙ is element-wise multiplication. This produces a channel-wise attention vector w that rescales each feature map according to its importance for the classification task.

#### 3.2.2. Cross-Gated Fusion

To merge the outputs of both branches, a cross-gated fusion (CGF) block is used. Unlike simple concatenation, CGF learns a pair of complementary gates that control the contribution of each branch, enabling adaptive feature exchange between the semantic and local representations [63,64,65,66]. Given the descriptors $d_{A}$ and $d_{B}$, the fusion mechanism is defined as:

(5) $$g=\sigma (W_{g}[d_{A};d_{B}]+b_{g}),\;\;\;\;\;\;\;\tilde{g}=1-g$$

where g is a gating vector between 0 and 1, and $\left[d_{A};d_{B}\right]$ denotes concatenation. The complementary gate $\tilde{g}$ ensures that both branches retain proportional influence. The fused representation h is computed as:

(6) $$h=(g⊙d_{A})+(\tilde{g}⊙d_{B})+W_{h}tanh(W_{A}d_{A})⊙tanh(W_{B}d_{B})$$

In this expression, the first two terms represent gated linear combinations of each descriptor, while the third introduces a bilinear interaction that captures non-linear dependencies between branches.

The weights $W_{A}$, $W_{B}$, and $W_{h}$ are learnable projection matrices, and tanh (·) introduces controlled non-linearity to prevent activation saturation. Finally, dropout is applied to h to reduce overfitting. This mechanism encourages complementary learning: EfficientNetV2-B0 contributes macro-level structure, while MobileNetV3-Small reinforces micro-pattern recognition.

#### 3.2.3. Hybrid Descriptor and Classification Head

The fused representation h undergoes normalization and regularization before classification:

(7) $$z=Dropout(BN(h)),\hat{y}=Softmax(W_{c}z+b_{c})$$

where BN denotes batch normalization, which stabilizes feature distributions across mini-batches, and dropout introduces random feature deactivation to enhance generalization [67]. The final softmax layer outputs class probabilities $\hat{y}$ for each diagnostic category. Training minimizes the categorical cross-entropy loss with optional label smoothing ε:

(8) $$L=-\sum_{c=1}^{c}y_{c}\log\hat{y_{c}}(1-\varepsilon )+\frac{\varepsilon }{c}$$

where $y_{c}$ and ${\hat{y}}_{c}$ are the true and predicted probabilities for class c. Label smoothing mitigates overconfidence in predictions and encourages more calibrated probabilities. Optimization employs the AdamW algorithm, which decouples weight decay from gradient updates to improve generalization [68]. The learning rate follows a Warmup–Cosine schedule:

(9) $$\eta_{t}=\left\{\begin{array}{cc} \eta_{max}\frac{t}{T_{w}}, & t<T_{w}\\ \frac{1}{2}\eta_{max}\left[1+cos(\pi \frac{t-T_{w}}{T-T_{w}})\right] & t\geq T_{w} \end{array}\right\}$$

where $\eta_{t}$ is the learning rate at step t, $\eta_{max}$ is the maximum rate reached after the warmup period, $T_{w}$ is the number of warmup steps, and T is the total training duration.

This schedule allows gradual learning rate growth during early epochs to avoid unstable updates, followed by smooth decay to favor fine convergence [69,70]. In practice, this combination produced stable optimization and low GPU memory usage.

### 3.3. Visual Saliency Maps with Grad-CAM

To enhance model interpretability and better understand the decision process, Gradient-weighted Class Activation Mapping (Grad-CAM) was employed. This method highlights the spatial regions within an image that most influence the model’s prediction. Grad-CAM is especially effective for convolutional architectures, where visual explanations are essential for assessing robustness and clinical reliability [33,42,43,71].

Formally, let $y_{c}$ represent the pre-softmax score corresponding to class c, and $A^{k}$ the k-th feature map from the last convolutional layer, indexed by spatial coordinates (i,j).

*Grad*-CAM computes the gradient of $y_{c}$ with respect to each activation $A_{ij}^{k}$, thereby tracing class-specific information back through the network. These gradients are globally averaged over spatial dimensions to obtain importance weights $\alpha_{c}^{k}$:

(10) $$\alpha_{c}^{k}=\frac{1}{Z}\sum_{i}\sum_{j}\frac{\partial y_{c}}{\partial A_{ij}^{k}}$$

where Z=H×W denotes the total number of spatial locations in the feature map. The coefficient $\alpha_{c}^{k}$ quantifies the relative relevance of the feature map $A^{k}$ to the prediction of class c. The class-discriminative saliency map $L_{c}^{\mathrm{Grad}\text{-}\mathrm{CAM}}\in R^{u\times v}$ is then generated by a weighted combination of the activation maps, followed by a rectified linear unit:

(11) $$L_{c}^{Grad-CAM}=ReLU\left(\sum_{k}\alpha_{c}^{k}A^{k}\right)$$

where *ReLU* is defined as:

(12) $$ReLU(x)=\left\{\begin{array}{cc} x, & x>0,\\ 0, & x\leq 0, \end{array}\right\}$$

This operation ensures that only positive contributions supporting the predicted class are preserved, effectively emphasizing the most discriminative regions of the image that drive the model’s final decision [71,72,73]. As a result, Grad-CAM provides an intuitive visualization of the network’s attention, reinforcing interpretability and confidence in model predictions.

## 4. Results and Discussion

The proposed BiLight-Attn-LC network was implemented in Python 3.13 using TensorFlow 2.14 and Keras 3.11.3. Experiments were executed on a workstation equipped with an Intel i9-13900KF CPU (3.0 GHz, 64 GB RAM), an NVIDIA RTX A4500 GPU (20 GB VRAM), and a 1 TB PCIe SSD. All computations employed mixed-precision (float16) training and dynamic GPU memory growth to optimize VRAM usage. The model required an average of 106 s per epoch and achieved an inference time of 101 ms per test step (≈6.3 ms per image). The network comprises 7,666,127 parameters in total (807,439 trainable and 6,858,688 non-trainable), corresponding to a model size of 29.24 MB.

The dataset was split into 80% training, 10% validation, and 10% testing, preserving reproducibility via a fixed random seed. Images were resized to 224 × 224 RGB and loaded through Keras ImageDataGenerator with a batch size of 16, enabling shuffling for training and validation only. A soft H&E jitter augmentation was applied to simulate staining variability (hue ± 6%, eosin ± 5%, density ± 4%, brightness ± 2%, contrast ± 3%), while MixUp and CutMix were disabled to preserve the morphological integrity of tissue structures. The network was optimized with AdamW (learning rate = 5 × 10^−4^, weight decay = 1 × 10^−4^) and categorical cross-entropy loss (label smoothing = 0.00). Learning-rate scheduling followed a Warm-Up + Cosine Decay policy, with 3 warm-up epochs and 100 total epochs, capped at 1500 steps per epoch to ensure stable GPU memory.

All convolutional backbones (EfficientNetV2-B0 and MobileNetV3-Small) were frozen during the first training phase to focus optimization on the fusion and attention modules. Regularization included batch normalization and dropout (0.30) in the classification head. Training used early stopping (patience = 8, restore_best_weights = True), ModelCheckpoint (on validation loss), and an epoch-end garbage-collection callback to prevent memory fragmentation. Model performance was assessed through standard multi-class metrics—accuracy, precision, recall, F1-score, Matthews Correlation Coefficient (MCC), and ROC-AUC—computed as follows [38,42,43,44,71,74]. The metrics were computed according to the following definitions:

(13) $$\mathrm{Accuracy}=\frac{TP+TN}{TP+TN+FP+FN}$$

(14) $$\mathrm{Precision}=\frac{TP}{TP+FP}$$

(15) $$\mathrm{Recall}=\frac{TP}{TP+FN}$$

(16) $$F_{1}score=2\cdot \frac{\mathrm{Precision}\cdot \mathrm{Recall}}{\mathrm{Precision}+\mathrm{Recall}}$$

(17) $$\mathrm{ROC}\;\mathrm{AUC}=\int_{0}^{1}\mathrm{TPR}\left(\mathrm{FPR}\right)\;d\left(\mathrm{FPR}\right)$$

(18) $$\mathrm{MCC}=\frac{\left(TP\cdot TN\right)-\left(FP\cdot FN\right)}{\sqrt{\left(TP+FP\right)\left(TP+FN\right)\left(TN+FP\right)\left(TN+FN\right)}}$$

where TP, TN, FP, and FN denote true positives, true negatives, false positives, and false negatives, respectively. TPR are True Positive Rate and FPR are False Positive Rate.

### 4.1. Results Analysis

Model performance was assessed on the test set, the training set, and on the entire dataset (all). Each histopathological tile in the LC25000 dataset corresponds to a single organ and a single diagnostic category, meaning that the model performs multi-class classification across five mutually exclusive classes (three lung-related and two colon-related). The BiLight-Attn-LC network does not assume or attempt to detect simultaneous malignancies from different organs within the same specimen; rather, each image patch is independently assigned to one of the five categories.

**Figure 3:**
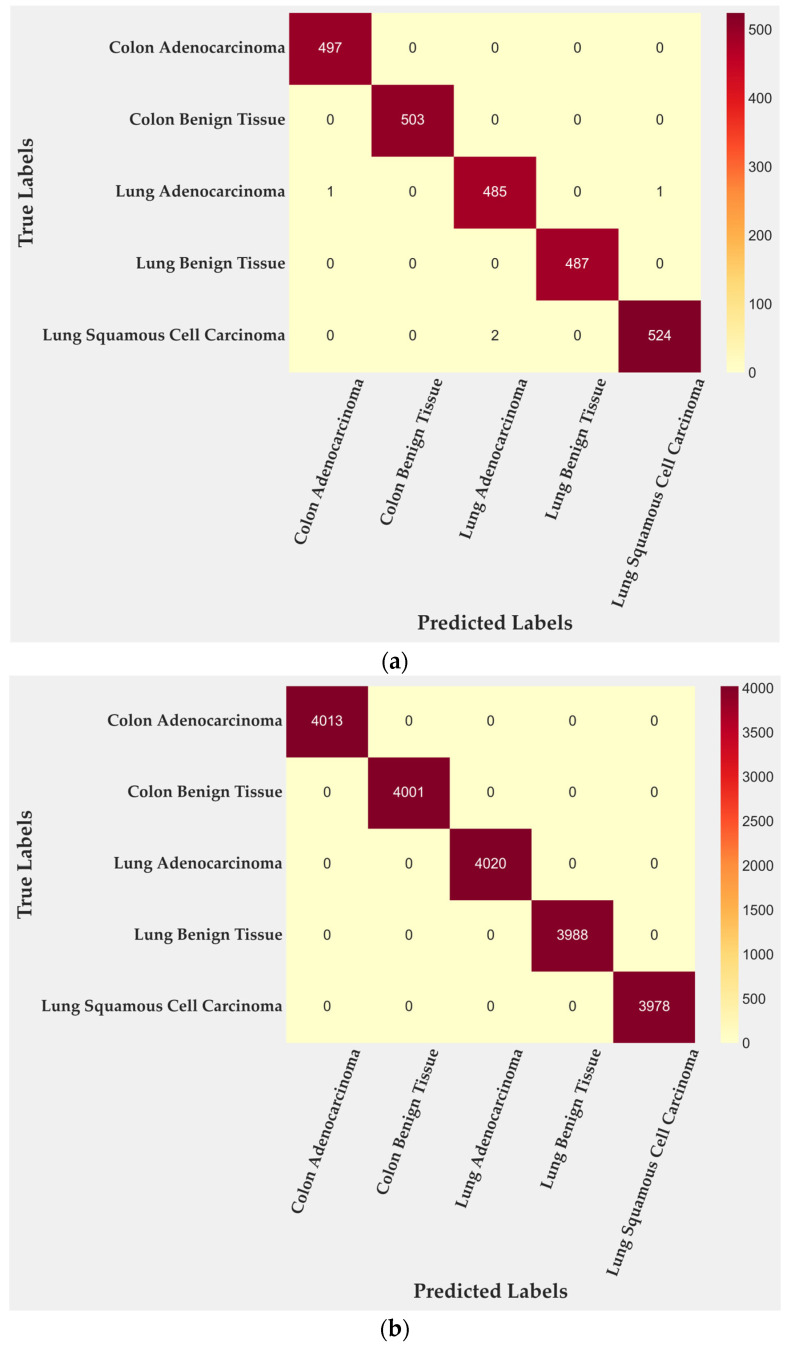

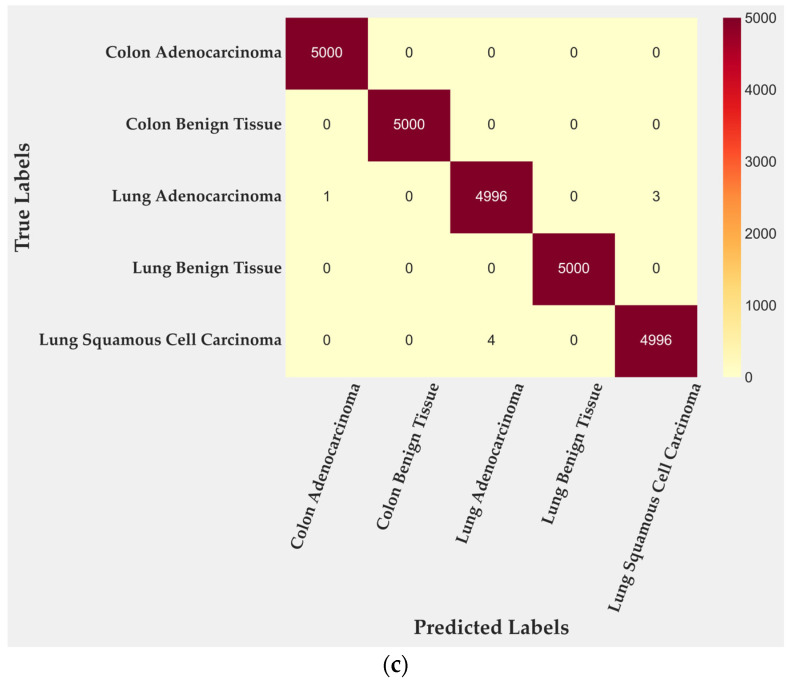
Confusion matrices for BiLight-Attn-LC across different evaluation settings. (**a**) Test set; (**b**) train set; (**c**) all dataset.

In the test subset, only four of 2500 tiles were misclassified, corresponding to 99.84% accuracy. All errors occurred within malignant lung classes: two tiles from lung adenocarcinoma and two from lung squamous cell carcinoma. Colon adenocarcinoma, colon benign tissue, and lung benign tissue were identified without error. To further quantify this behavior, class-level TPR and FPR were computed from the confusion matrix. Lung adenocarcinoma achieved a TPR of 0.996 with an FPR of 0.0000, while lung squamous cell carcinoma reached a TPR of 0.997 and an FPR of 0.0005. Training performance (Figure 3b) reached complete accuracy, confirming that the cross-gated fusion module captures subtle texture variations. Although perfect training results can suggest mild overfitting, the evaluation on the entire dataset (Figure 3c) reproduced the same error pattern, indicating that the learned representations remain stable rather than memorized. Only four misclassifications remained confined to malignant pulmonary samples, showing that the model generalizes well across folds and organ sites.

Class-wise metrics (Table 2) indicate that precision, recall and F1-score remain close to 1.0 for benign tissues and for Colon Adenocarcinoma. Slightly lower yet still near-perfect scores were observed in Lung Adenocarcinoma (F1-score ≈ 0.996) and Lung Squamous Cell Carcinoma (F1-score ≈ 0.971), which correspond to regions where acinar and keratinizing structures overlap microscopically. The variation therefore reflects biological heterogeneity more than algorithmic limitation.

Global indicators (Table 3) show accuracy, precision, recall, and F1-score at 0.9984 with an MCC of 0.9980 on the test set. Across the complete dataset, all metrics remain above 0.9996. The difference between training and test accuracy is 0.16%, confirming that the learned features retain discriminative power outside the training distribution.

**Figure 4:**
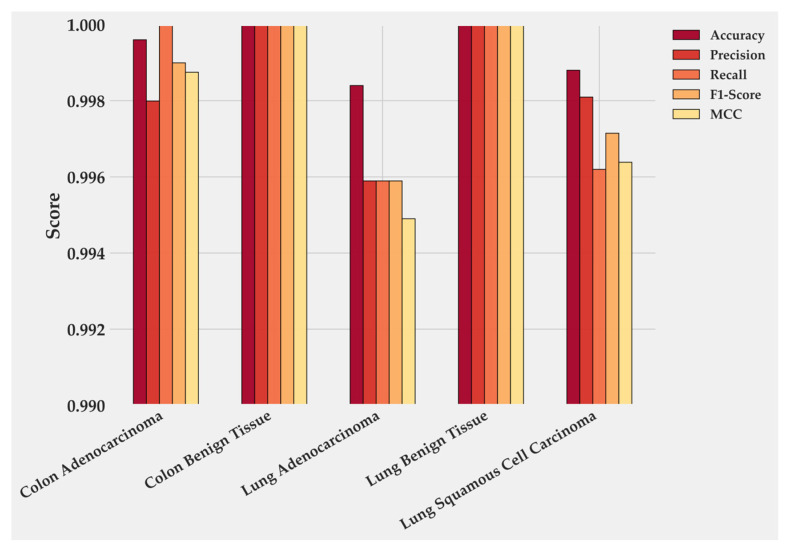
Class-wise performance of BiLight-Attn-LC on the test dataset.

Figure 4 quantifies these class-level results. Colon Benign Tissue and Lung Benign Tissue both reach perfect scores across all metrics. Colon Adenocarcinoma follows closely, with precision 0.9979 and F1-score 0.9989. Lung Adenocarcinoma maintains balanced precision and Recall near 0.996, producing the smallest gap between the two malignant categories. LUSC shows the lowest values: accuracy 0.9989, recall 0.9961, and F1-score 0.9714, which together account for its minor reduction in overall performance. These numbers mirror the confusion matrix and confirm that errors are rare and localized.

The observed strength aligns with the architectural design. The cross-gated fusion layer then reconciles the two descriptors and reduces spurious activations, which is consistent with the absence of errors in benign tissues and the very low error rate in malignant lung patches. Together, these choices explain why performance remains high across splits and why residual mistakes appear only where morphology is genuinely ambiguous. Benign tissues, with their regular glandular or alveolar organization, are easily distinguished. Malignant lung lesions, by contrast, present greater nuclear variability and irregular stroma, which introduce ambiguity at the patch level [35,36,37,38,39,40,41,42,43,44,45].

**Figure 5:**
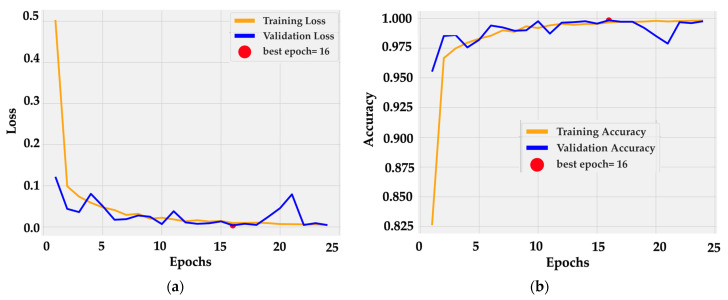
Training dynamics of BiLight-Attn-LC over 25 epochs. (**a**) Training and validation loss curves; (**b**) Training and validation accuracy curves.

Training dynamics were stable and efficient. The loss curve (Figure 5a) dropped sharply within the first three epochs, from 0.5 to below 0.05, and then converged smoothly toward zero. Validation loss followed a similar path with small oscillations around 0.02 after epoch 10. The minimum was reached at epoch 16, marked as the optimal checkpoint.

Accuracy curves (Figure 5b) confirm rapid convergence: training accuracy rose from 0.82 to 0.99 by epoch 5 and reached 1.00 near epoch 16. Validation accuracy tracked the same trajectory, fluctuating slightly around 0.99–1.00 without divergence. The near overlap between curves indicates consistent generalization and the absence of overfitting. This behavior is consistent with the architectural design, in which frozen dual backbones provide stable low-level features while the cross-gated fusion layer adapts high-level semantics with minimal parameter drift.

**Figure 6:**
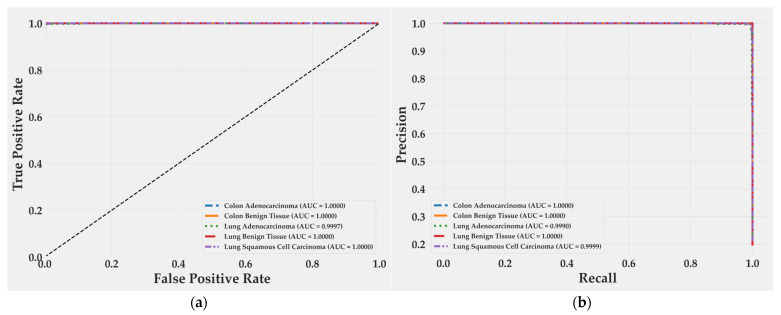
ROC and precision–recall curves for BiLight-Attn-LC on the test set. (**a**) Receiver operating characteristic curves; (**b**) Precision-Recall curves.

Receiver operating characteristic curves (Figure 6a) show perfect separability for almost all classes, with area under the curve (AUC) values of 1.0000 for Colon Adenocarcinoma, Colon Benign Tissue, Lung Benign Tissue, and Lung Squamous Cell Carcinoma. Lung Adenocarcinoma achieved 0.9997, a negligible difference of 0.03%. Precision–recall curves (Figure 6b) follow the same trend: all classes reach an AUC of 1.0000 except Lung Adenocarcinoma (0.9990) and Lung Squamous Cell Carcinoma (0.9999). Both remain at the upper bound of discriminative performance, confirming that false positives and false negatives are practically absent. These results reflect an extremely well-calibrated decision boundary and a model that preserves recall without sacrificing precision, even in morphologically overlapping subtypes.

### 4.2. Visual Saliency Maps for Model Explainability

**Figure 7:**
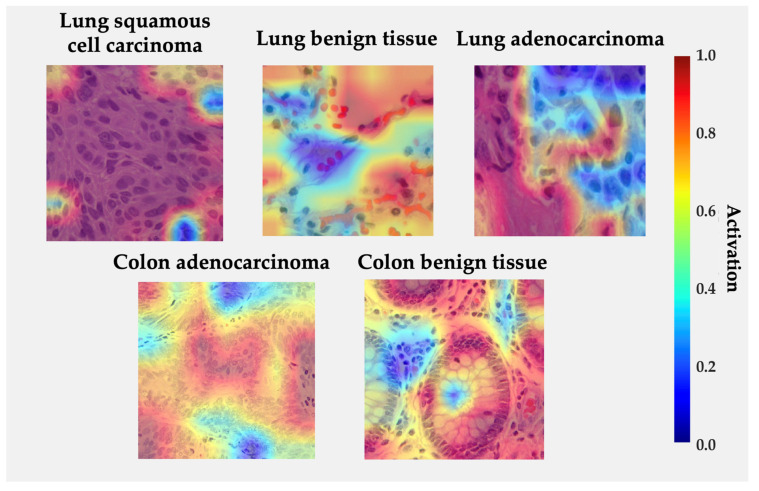
Grad-CAM visualizations for each class from LC25000 dataset. Regions in red indicate high model activation, while blue indicates low activation.

Figure 7 displays Grad-CAM activation overlays generated for representative histological samples from each class. The color intensity reflects the relative contribution of local regions to the model’s decision. The visualization reveals that the proposed model focuses on morphologically meaningful structures rather than background artifacts.

In lung squamous cell carcinoma, activations concentrate over clusters of polygonal cells with dense eosinophilic cytoplasm and keratin pearls, consistent with the histologic hallmarks of squamous differentiation. For lung adenocarcinoma, high activations are observed along glandular or acinar formations and nuclear crowding areas, indicating that the network correctly attends to atypical epithelial arrangements. In benign lung tissue, activations are diffuse and low-intensity, reflecting the absence of neoplastic patterns. Similarly, in colon adenocarcinoma, the model highlights irregular glandular borders and hyperchromatic nuclei located at the invasive front, while benign colon tissue exhibits localized responses restricted to the epithelial lining and lumenal boundaries, suggesting recognition of normal crypt architecture. The absence of strong activations in normal samples and the spatial agreement between attention peaks and diagnostically relevant regions reinforce the model’s interpretability and biological plausibility [33,42,43,51,58].

### 4.3. Discussion

As shown in Table 4, the proposed BiLight-Attn-LC reached 99.84% in accuracy, precision, recall, and F1-score on the LC25000 dataset. This result is only 0.12% below AdBet-WOA (99.96%) [46], yet the latter relies on a hybrid meta-heuristic optimization pipeline that combines multiple CNN embeddings, Whale Optimization, and an external SVM classifier. Such architectures require heavy feature extraction, feature reduction, and repeated tuning cycles, consuming substantial computational resources. BiLight-Attn-LC achieves nearly identical accuracy through a single, memory-safe training process that avoids handcrafted descriptors, external feature selectors, and meta-heuristic optimizers. The gain is practical rather than marginal: comparable accuracy with far lower complexity and full reproducibility.

Relative to single-backbone transfer models such as EfficientNetB3 [43] or Inception-ResNetV2 [44], the proposed dual-encoder scheme yields measurable benefits. EfficientNetV2-B0 captures global organization and tissue semantics, while MobileNetV3-Small preserves micro-textural information critical for differentiating subtle malignancies. The cross-gated fusion layer balances these complementary embeddings dynamically, preserving relevant cues while filtering redundant activations. This integration explains both the small but consistent performance gain (0.4–3.9%) and the smooth convergence behavior observed during training.

Compared with attention-intensive networks such as LMVT [51] or EfficientNetV2-L [33], which achieve similar accuracy at the cost of high VRAM usage and multi-stage fine-tuning, BiLight-Attn-LC maintains contextual reasoning with a compact architecture. The near-perfect ROC and PR curves demonstrate that the model learns well-calibrated decision boundaries even in histologically ambiguous regions like lung adenocarcinoma versus squamous cell carcinoma.

The principal strengths of BiLight-Attn-LC lie in its end-to-end design, interpretability, and computational efficiency. It avoids complex post hoc optimizers, handcrafted features, or auxiliary classifiers, ensuring transparency and reproducibility. Grad-CAM maps highlight diagnostically meaningful areas—acinar borders, keratin pearls, and glandular irregularities—supporting the model’s alignment with human diagnostic reasoning [33,42,43,51,58].

Nonetheless, limitations remain. The LC25000 dataset includes 1250 original slides (750 lung and 500 colon) expanded to 25,000 augmented tiles. While this ensures class balance, it limits morphological diversity and may overestimate generalization due to repeated color or texture patterns. Some studies report near-perfect performance on LC25000, including models achieving 100% accuracy [53]; however, these results rely solely on intra-dataset evaluation without external validation, which limits methodological comparability and may not generalize across institutions with different staining or acquisition conditions.

Future work will focus on external and collaborative validation. We plan to extend experiments using histological images from the National Cancer Institute GDC Data Portal, emphasizing benign and morphologically complex malignant tissues [38,43,44]. Beyond accuracy, our goal is to evaluate diagnostic reliability under three decision modalities: AI-only, pathologist-only, and pathologist–AI combined. This framework will quantify how model suggestions influence human decision-making and whether human–AI consensus improves diagnostic confidence or introduces bias. A quantitative Grad-CAM assessment will complement this by measuring spatial agreement between model attention and expert-marked diagnostic regions, enabling objective interpretability scoring.

In sum, BiLight-Attn-LC achieves state-of-the-art accuracy with substantially reduced computational burden, while offering interpretability and design simplicity suitable for clinical integration. The next phase moves beyond metrics toward understanding how the model and the clinician interact bridging algorithmic precision with human diagnostic judgment.

## 5. Conclusions

The proposed BiLight-Attn-LC network shows that high diagnostic accuracy can be achieved without relying on complex or computationally expensive architectures. By integrating EfficientNetV2-B0 and MobileNetV3-Small through a cross-gated attention mechanism, the model fuses global context and fine structural detail within a single, end-to-end training framework. This design removes the need for handcrafted descriptors, feature extraction, feature reduction, or metaheuristic optimization, maintaining efficiency and reproducibility.

On the LC25000 dataset, BiLight-Attn-LC reached 99.84% in accuracy, precision, recall, and F1-score, confirming its reliability in differentiating benign and malignant tissues of lung and colon origin. Grad-CAM visualizations confirmed that the model attends to histopathological structures relevant to expert diagnosis, supporting its interpretability and clinical potential.

Future work will extend evaluation to multi-institutional datasets and involve joint assessments between AI and human experts. A comparative framework including AI-only, human-only, and human–AI decisions will be implemented to measure diagnostic bias and trust in assisted interpretation. The goal is to move beyond performance metrics toward understanding how human reasoning and algorithmic prediction can converge in reliable, clinically aligned decision-making.

## Figures and Tables

**Table 1:** Summary of the LC25000 dataset used in this study.

Class	Description	Number of Images
Lung benign tissue	Normal pulmonary parenchyma	5000
Lung adenocarcinoma	Malignant gland-forming epithelium	5000
Lung squamous cell carcinoma	Keratinizing malignant epithelium of bronchial origin	5000
Colon adenocarcinoma	Dysplastic glandular proliferation in colonic mucosa	5000
Colon benign tissue	Normal colonic mucosa without neoplastic changes	5000

**Table 2:** Class-wise performance (Precision, Recall, and F1-score) for the test, training, and full dataset.

Dataset	Class	Precision	Recall	F1-Score	Accuracy	MCC
Test	Colon Adenocarcinoma	0.9979	1.000	0.9989	0.9996	0.9987
	Colon Benign Tissue	1.000	1.000	1.000	1.000	1.000
	Lung Adenocarcinoma	0.9958	0.9958	0.9958	0.9984	0.9948
	Lung Benign Tissue	1.000	1.000	1.000	1.000	1.000
	Lung Squamous Cell Carcinoma	0.9989	0.9961	0.9714	0.9988	0.9963
Train	Colon Adenocarcinoma	1.000	1.000	1.000	1.000	1.000
	Colon Benign Tissue	1.000	1.000	1.000	1.000	1.000
	Lung Adenocarcinoma	1.000	1.000	1.000	1.000	1.000
	Lung Benign Tissue	1.000	1.000	1.000	1.000	1.000
	Lung Squamous Cell Carcinoma	1.000	1.000	1.000	1.000	1.000
All	Colon Adenocarcinoma	0.9998	1.000	0.9999	0.9999	0.9998
	Colon Benign Tissue	1.000	1.000	1.000	1.000	1.000
	Lung Adenocarcinoma	0.9992	0.9992	0.9992	0.9996	0.9990
	Lung Benign Tissue	1.000	1.000	1.000	1.000	1.000
	Lung Squamous Cell Carcinoma	0.9993	0.9992	0.9992	0.9997	0.9991

**Table 3:** Global evaluation metrics for the test, training, and full dataset.

Dataset	Images	Accuracy	Precision	Recall	F1-Score	MCC
Test	2500	0.9984	0.9984	0.9984	0.9984	0.9980
Train	20,000	1.000	1.000	1.000	1.000	1.000
All	25,000	0.9997	0.9997	0.9997	0.9997	0.9996

**Table 4:** Comparative BiLight-Attn-LC architecture with recent algorithms.

Methods	Dataset Used	Accuracy (%)	Precision (%)	Recall (%)	F1-Score (%)
EfficientNetB3 [43]	LC25000 + National Cancer Institute GDC Data Portal	99.39	99.39	99.39	99.39
InceptionResNetV2 [44]	LC25000 + National Cancer Institute GDC Data Portal	95.90	95.91	95.90	95.89
Pre-trained DL models with KELM [45]	Gland Segmentation in Colon Histology Images and LC25000	98.9	96.7	95.8	97.6
AdBet-WOA [46]	LC25000	99.96	99.96	99.97	99.96
MPADL-LC3 [48]	LC25000	99.27	98.18	98.17	98.17
BERTL-HIALCCD [49]	LC25000	99.22	98.07	98.06	98.06
HIELCC-EDL [50]	LC25000	99.60	99.00	99.00	99.00
LMVT [51]	LC25000	99.75	-	99.61	99.44
ViT-DCNN [52]	LC25000	94.24	94.37	94.24	94.23
EffcientNetV2-L [33]	LC25000	99.97	-	-	99.97
1-D CNN with Squeeze-and-Excitation [53]	LC25000	100	100	100	100
CNN embeddings with handcrafted descriptors [54]	LC25000	99.70	99.70	99.70	99.70
Multiscale Deep Features Integration of Compact CNN [55]	LC25000	99.78	99.78	99.78	99.78
Lightweight Multi-Scale CNN [57]	LC25000	99.20	99.16	99.36	99.16
MEGWO-LCCHC with DNN [38]	LC25000 + National Cancer Institute GDC Data Portal	94.8	94.81	94.8	94.8
BiLight-Attn-LC (Proposed)	LC25000	99.84	99.84	99.84	99.84

## Data Availability

The data presented in this study are available in Kaggle at https://www.kaggle.com/datasets/andrewmvd/lung-and-colon-cancer-histopathological-images (accessed on 6 October 2025), reference number https://doi.org/10.48550/arXiv.1912.12142. These data were derived from the following resources available in the public domain: Lung and Colon Cancer Histopathological Image Dataset (LC25000)—arXiv:1912.12142—https://academictorrents.com/details/7a638ed187a6180fd6e464b3666a6ea0499af4af (accessed on 6 October 2025).

## Abbreviations

AI	Artificial Intelligence
CAD	Computer-Aided Diagnosis
CNN	Convolutional Neural Network
DL	Deep Learning
ECA	Efficient Channel Attention
GeM	Generalized Mean Pooling
GAP	Global Average Pooling
GMP	Global Max Pooling
CGF	Cross-Gated Fusion block
BN	Batch Normalization
LN	Layer Normalization
ReLU	Rectified Linear Unit
Grad-CAM	Gradient-weighted Class Activation Mapping
MCC	Matthews Correlation Coefficient
ROC	Receiver Operating Characteristic
PR	Precision–Recall
AUC	Area Under the Curve
H&E	Hematoxylin and Eosin staining
KELM	Kernel Extreme Learning Machine
SVM	Support Vector Machine
AdBet-WOA	Adaptive β-Hill Climbing Whale Optimization Algorithm
MPADL-LC3	Marine Predators Algorithm Deep Learning model for LC25000
BERTL-HIALCCD	BERT-like Hybrid Integrated Attention Lung–Colon Cancer Detector
HIELCC-EDL	Hybrid Ensemble Lung–Colon Cancer Classifier based on Deep Learning
LMVT	Lightweight Multi-Vision Transformer
ViT-DCNN	Vision Transformer with Deformable Convolutional Neural Network
BiLight-Attn-LC	Proposed Bidirectional Lightweight Attention Network for Lung and Colon Classification
AdamW	Adaptive Moment Estimation with Decoupled Weight Decay Optimizer
VRAM	Video Random Access Memory
TPU/GPU	Tensor Processing Unit/Graphics Processing Unit
TP	True Positives
TN	True Negatives
FP	False Positives
FN	False Negatives
TPR	True Positive Rate
FPR	False Positive Rate
